# Supplementary material for: Type-2 innate lymphoid cells control the development of atherosclerosis in mice
Source: Nat Commun. 2017 Jun 7;8:15781. doi: 10.1038/ncomms15781 (PMC5467269; doi:10.1038/ncomms15781)
Supplement: Supplementary Information — Supplementary Figures. [file ncomms15781-s1.docx]

Supplementary Figure 1


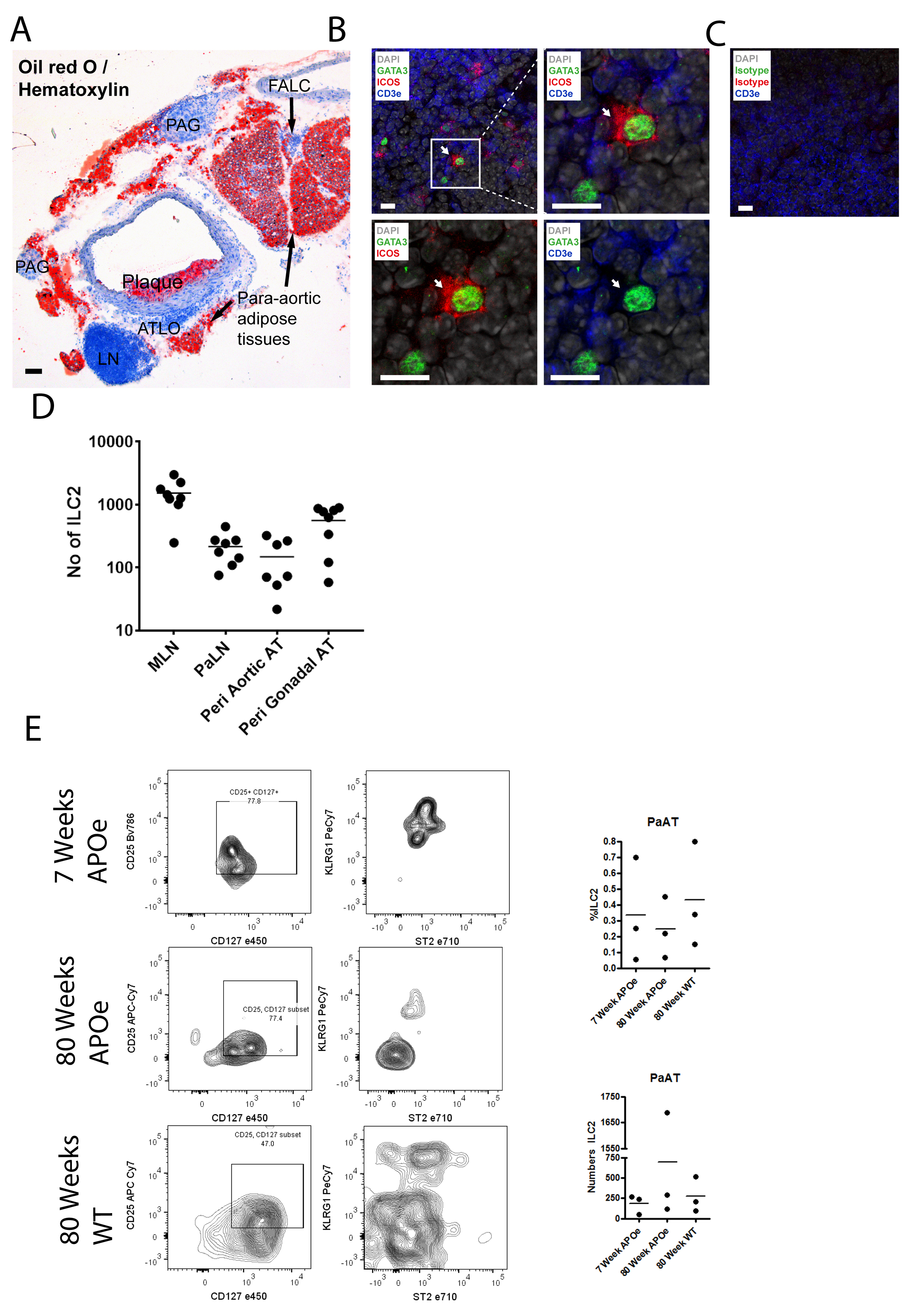


**Supplementary Figure 1. ILC2 are present in para-aortic adipose tissue and fat-associated lymphoid clusters around the aorta of *Apoe^-/-^* mice.**

(**A**) Hematoxylin and Oil red O staining show presence of para-aortic lymph nodes (LN), artery tertiary lymphoid organs (ATLO) and fat-associated lymphoid clusters (FALC) in para aortic adipose tissue (scale bar 100µm). Para-aortic ganglions (PAG) are also shown. (**B, C**) Immunoflorescence of CD3^-^ GATA3^+^ ICOS^+^ ILC2 in lymph node of young *Apoe^-/-^* mice (**B**, isotype control **C**, scale bar 10µm). (**D**) ILC2 numbers were calculated in mesenteric and para aortic lymph nodes (MLN and PaLN) and compared to para aortic (PaAT) and peri gonadal adipose tissue in 20+ week old *Apoe^-/-^* mice. (**E**) Surface phenotype of ILC2 in young *Apoe^-/-^*, aged *Apoe^-/-^* and WT. The proportion of ILC2 in total PaAT remains unchanged during ageing (80+ weeks) of *Apoe^-/-^* mice on chow diet and does not differ in WT of the same age. Graph data points represent individual mice.

**Supplementary Figure 2. ILC2 numbers in aorta, PaAT and peripheral lymph nodes in young and aged mice** (**A**) ILC2 present in aged *Apoe^-/-^* ATLO are morphologically similar to those observed in peripheral lymph nodes (scale bar 10µm). (**B**) Flow cytometric analysis confirms para-aortic ATLO contain Lineage^-^ CD45^+^ ICOS^+^ CD25^+^ CD127^+^ ILC2. ILC2 in spleen are shown for comparison. (**C**) The para-aortic FALC, similar to those found at other sites, contain proliferating immune cells and functional lymph and blood vasculature (scale bar 100µm). **(D)** Decreased recovery of ILC2 from spleen during high fat feeding (n=7) compared to normal chow (N=6). **(E)** Typical post sorting cell purity**.** Statistical significance was determined by Mann-Whitney U test.


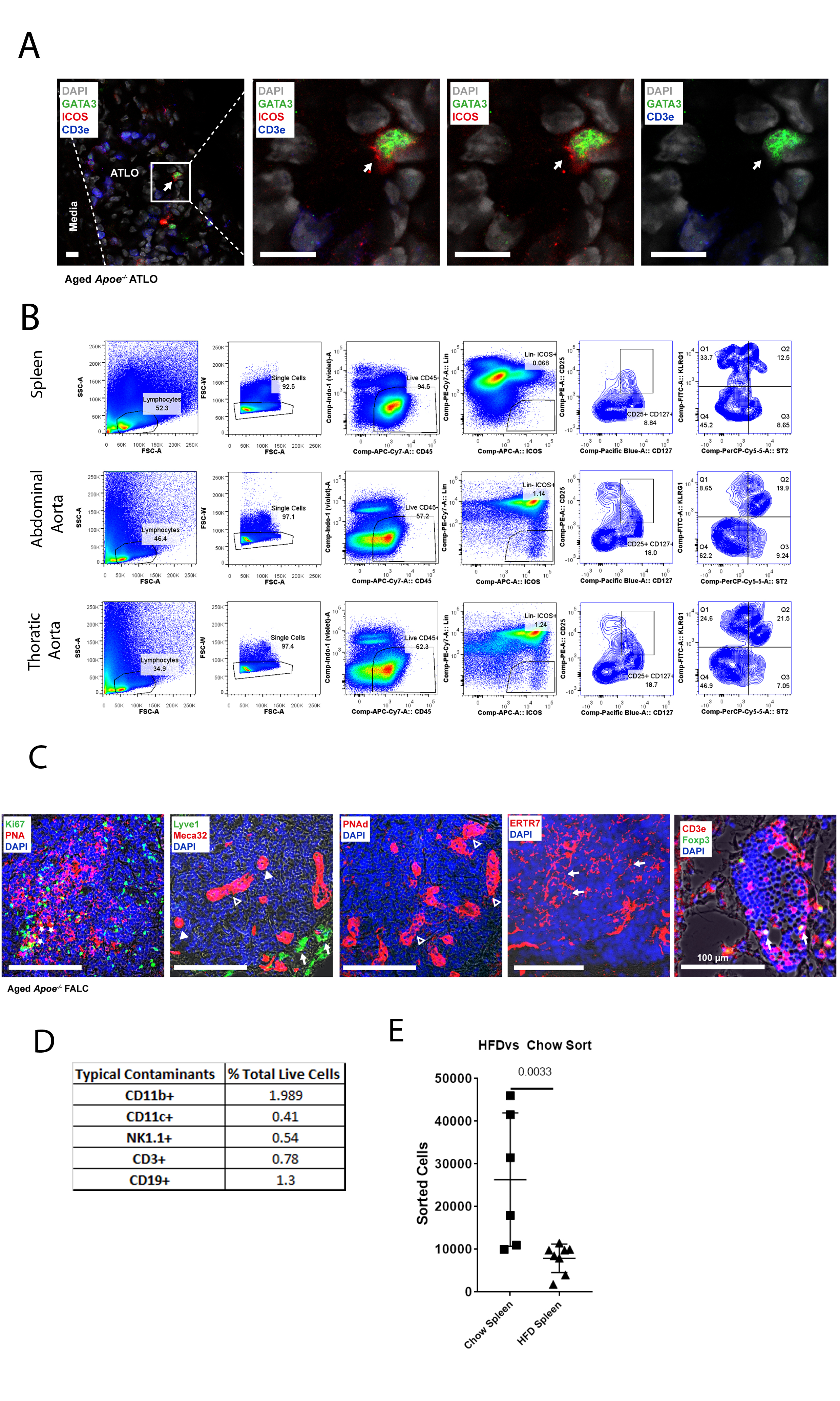


Supplementary Figure 3:


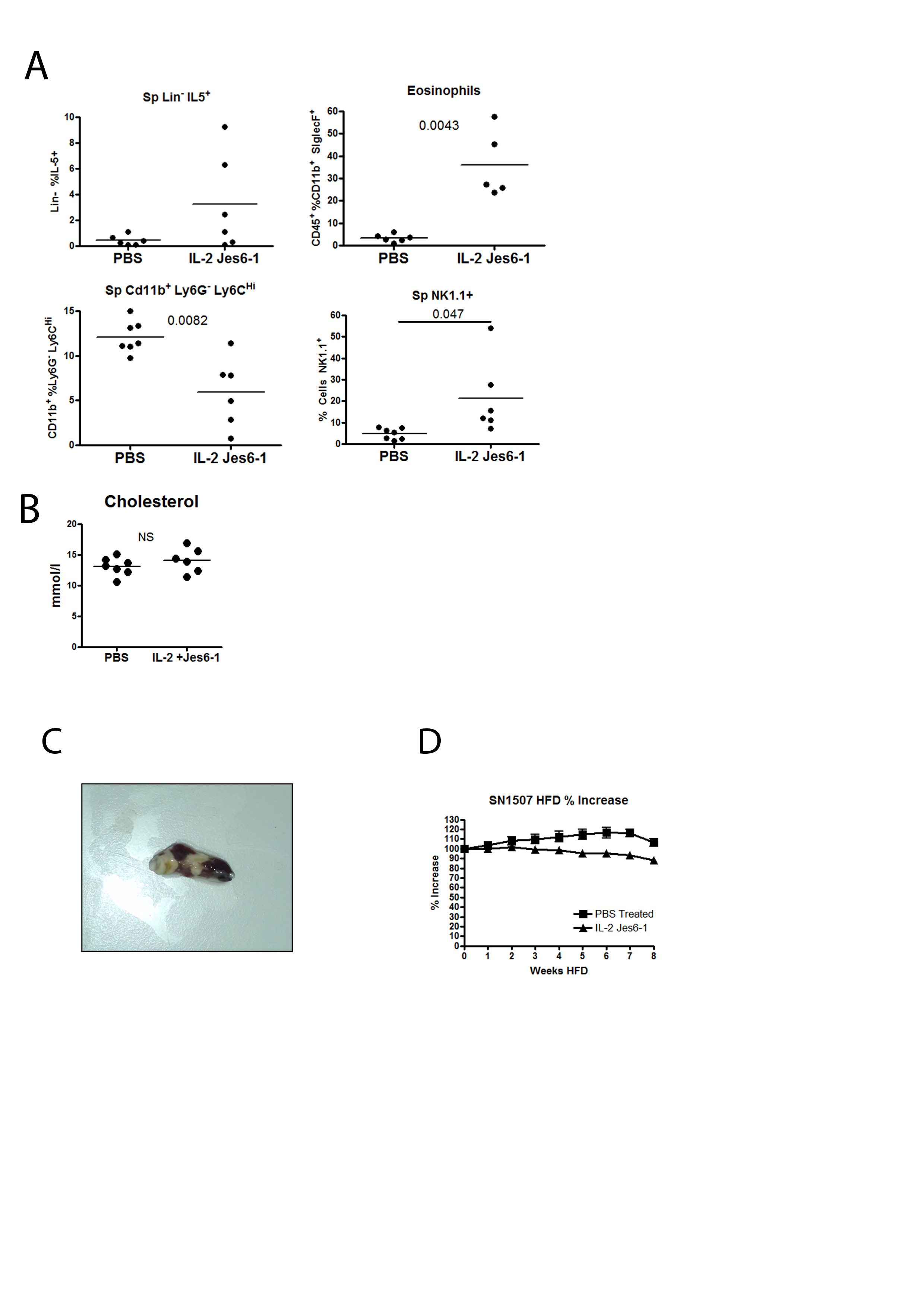


**Supplementary Figure 3. Phenotypic changes in IL-2/Jes6-1 treated mice.** *Apoe^-/-^/Rag2^-/-^* mice undergoing prolonged treatment with IL-2/Jes6-1 complexes have an expanded population of IL-5^+^ ILC2 (Lin^-^ ICOS^+^) and eosinophils and decreased CD11b^+^ Ly6G^-^ Ly6c^hi^ inflammatory monocytes (**A**). There was no difference in the amount of cholesterol present in the serum of treated mice (**B**) However, the spleens of treated mice showed considerable fibrosis (**C**) and the mice experienced weight loss (**D**). Graph data points represent individual mice. Statistical significance was determined by Mann-Whitney U test.

Supplementary Figure 4


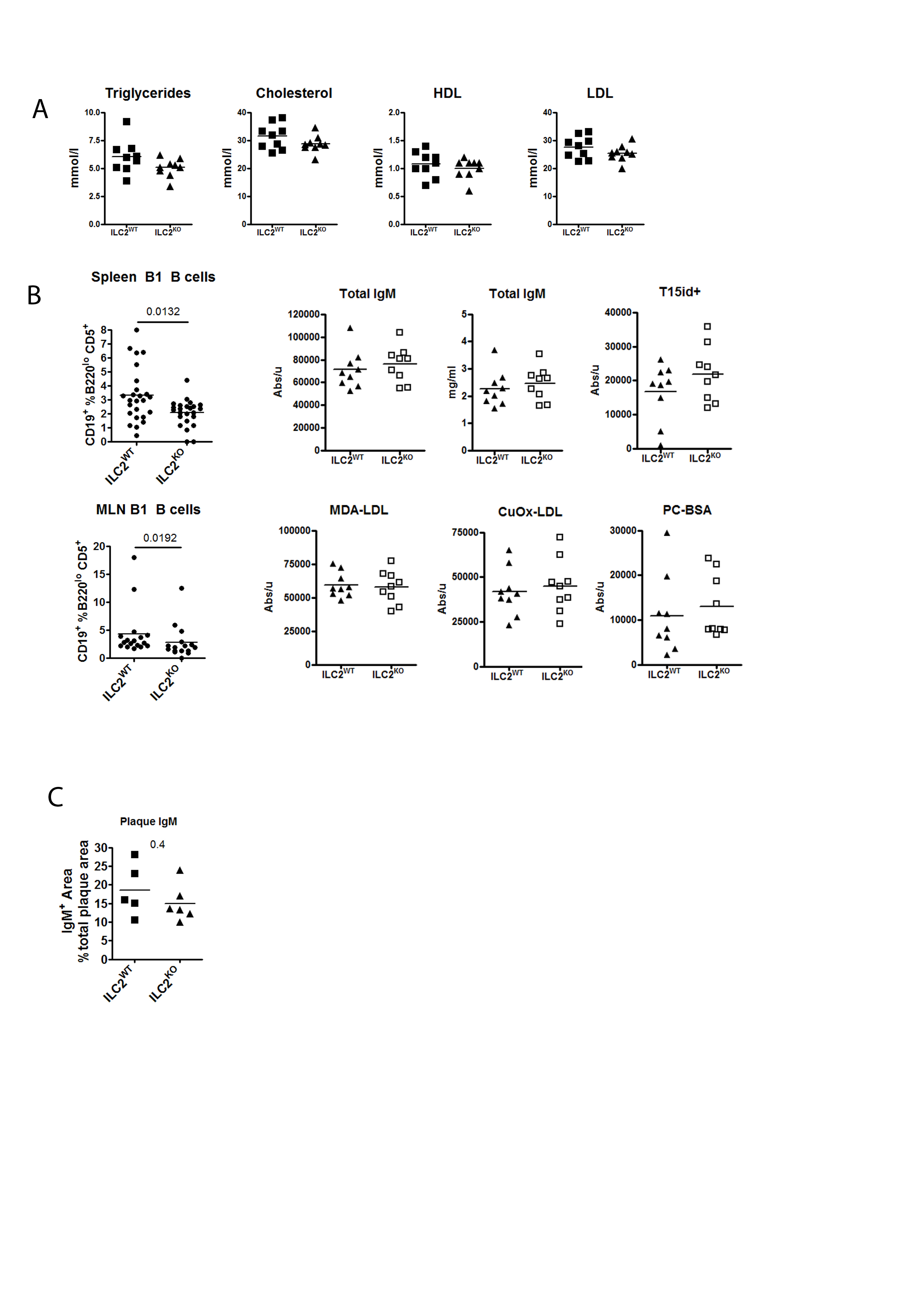


**Supplementary Figure 4.** **Serum biochemistry is unaltered in ILC2^KO^ mice and no role for B1 B cells**. (**A**) Triglycerides, cholesterol and HDL/LDL-cholesterol were measured in serum collected 8 weeks HFD. (**B**) Splenic and lymph node B1a B cells were deficient in ILC2^KO^ mice compared to ILC2^WT^. No changes in natural IgM antibodies were observed in the serum of ILC2^KO^ mice. (**C**) Plaque IgM deposition in the aortic sinus. Graph data points represent individual mice. Statistical significance was determined by Mann-Whitney U test.

Supplementary Figure 5


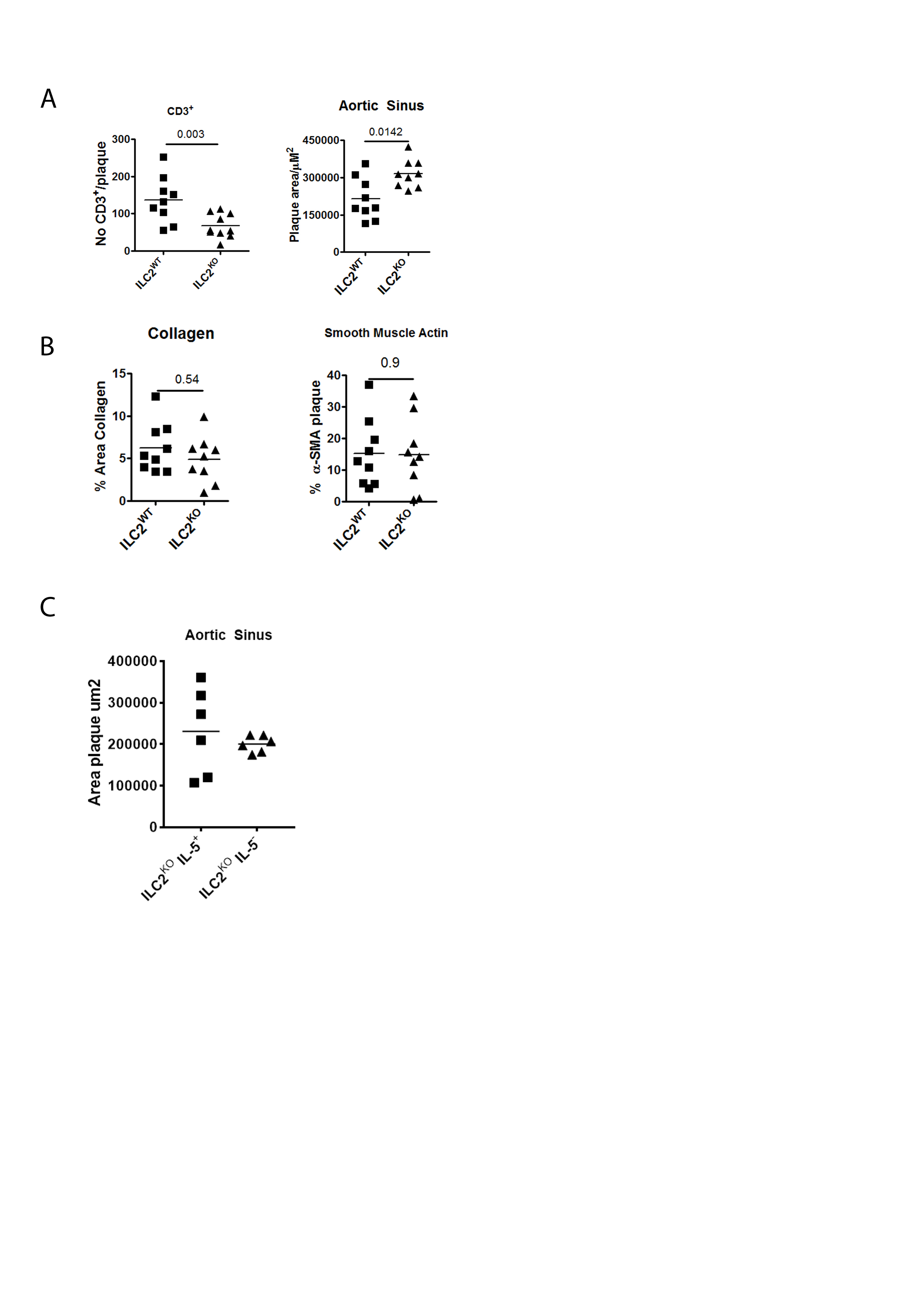


**Supplementary Figure 5. ILC2 alter plaque composition and ILC2-derived IL-5 and IL-13 are required to reduce atherosclerosis.**

Increased atherosclerotic lesion size but decreased CD3^+^ cells in aortic sinus of ILC2^KO^ recipients (**A**). Collagen and smooth muscle actin in the aortic sinus of ILC2^KO^ mice (**B**). Oil red O staining of aortic sinus after bone marrow transplant with ILC2^KO^ IL-5^-^ donor bone marrow n= 6 mice per group (**C**). Graph data points represent individual mice. Statistical significance was determined by Mann-Whitney U test.
